# Low-dose penicillin exposure in early life decreases Th17 and the susceptibility to DSS colitis in mice through gut microbiota modification

**DOI:** 10.1038/srep43662

**Published:** 2017-03-08

**Authors:** Shuang Jin, Di Zhao, Chenwen Cai, Dongjuan Song, Jun Shen, Antao Xu, Yuqi Qiao, Zhihua Ran, Qing Zheng

**Affiliations:** 1 Division of Gastroenterology and Hepatology, Key Laboratory of Gastroenterology and Hepatology, Ministry of Health, Shanghai Inflammatory Bowel Disease Research Center, Renji Hospital, School of Medicine, Shanghai Jiao-Tong University, Shanghai Institute of Digestive Disease, 145 Middle Shandong Road, Shanghai 200001, China

## Abstract

Antibiotic exposure in early life can lead to a significant change of the gut microbiota and may contribute to later onset of inflammatory bowel disease (IBD). However, the relationship between early-life antibiotic treatment and IBD is ambiguous, according to contradicting results of epidemiologic studies. In the present study, we demonstrated that low-dose penicillin pre-treatment had a unique protective effect against mouse colitis induced by dextran sodium sulfate (DSS). Low-dose penicillin also suppressed the expression of pro-inflammatory cytokine IL-17 in various intestinal tissues, and decreased the amount of Th17 cells in small-intestine lamina propria. Neither metronidazole nor enrofloxacin had a similar effect. We further confirmed that low-dose penicillin could cause specific changes of the gut microbiota, especially the eradication of segmented filamentous bacteria (SFB). Mice without SFB inoculation showed no disparity when treated with penicillin or water. Taken together, the results showed that low-dose penicillin can achieve a highly specific manipulation of sensitive bacteria and interfere with development of intestinal immune system in early life. The study may further indicate the possibility of achieving a favorable immune state among a certain group of patients with IBD, or other autoimmune diseases, by fine-tuning the gut microbiota.

Inflammatory bowel disease (IBD) is a group of chronic, potentially disabling diseases of the gastrointestinal tract, mainly consisting of Crohn’s disease and ulcerative colitis. According to epidemiologic studies, the incidence of IBD is rising worldwide, especially in newly industrialized countries, while the mortality of the disease remains low^1^. This leaves the world with an ever-growing burden of IBD^1^. Apart from innovations in treatment optimization, study of the underlying mechanism of pathogenesis should also be part of the research priority. Hinted by the trend of incidence changes in the west and east, environmental factors including life style are thought to be associated with, or even responsible for the rising incidence of IBD^2^.

Antibiotic exposure in early life is among the environmental risk factors of IBD. But epidemiologic studies so far showed disparate data, indicating an important yet complicated role of it. A meta-analysis including several epidemiological reports from western countries showed a significant association between early-life antibiotic consumption and newly-onset Crohn’s disease, especially among children^3^. However, a recent study in Asia indicated an inverse association of antibiotic use and development of IBD^4^. Moreover, another survey found antibiotic consumption increased the risk of CD and UC among Caucasians but decreased the risk among Middle Eastern migrants^5^. This disparity warrants a need for basic science researches using animal models, which exclude many confounding factors in human real life.

Disturbance of gut microbiota has been proposed as the underlying mechanism of antibiotics’ effect on immune-mediating diseases, including IBD, asthma and obesity^6^,^7^. Recent years have witnessed the thriver of microbiota research as high-throughput sequencing technique substantially extended our knowledge of previous unculturable microorganisms. Plenty of researches have targeted the shifts of gut microbial composition during and after antibiotic treatment, both in human and animals^8^,^9^. Through the modification of gut microbiota, antibiotics may cause profound alterations of gut epithelium, immune cells and even intestinal neural system^10^,^11^. It might explain why and how antibiotic-caused microbiota changes in early life can impact IBD pathogenesis.

Most of the previous researches applied large doses of antibiotics or even cocktails. The modulatory effects of low-dose antibiotics on microbes have been overlooked while they were capable of altering the intestinal microbiota with lasting consequences on hosts^12^. Besides, antibiotic residues in foods and water supply have become an environment pollutant^13^, which causes an extra low-dose exposure to people including children apart from iatrogenic sources^14^. Thus, we aimed to answer whether and how early-life exposure to certain subtherapeutic antibiotics would change the susceptibility of IBD with a mouse model of experimental colitis. In the present study, we found low-dose penicillin had an unexpected protective effect against dextran sodium sulfate (DSS) induced colitis. We further examined the perturbation of gut microbiota and immune system by penicillin, and demonstrated the protection was dependent on the eradication of segmented filamentous bacteria (SFB) in the intestine.

## Results

### Low-dose penicillin exposure in early life has a protective effect against later DSS colitis in mice

**Figure 1:**
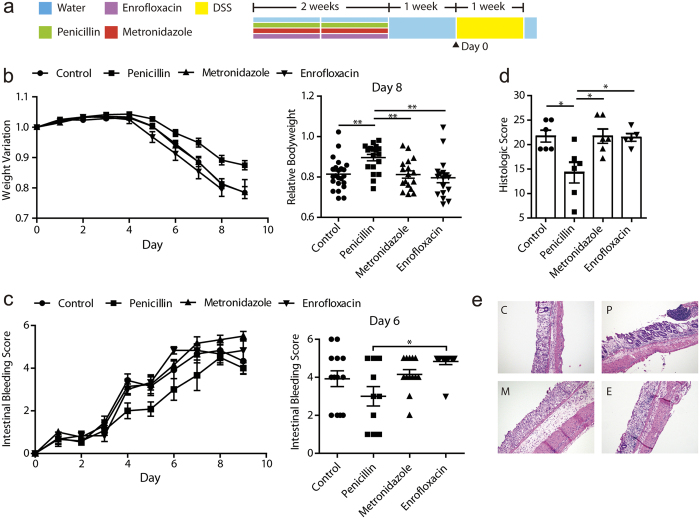
Effects of low-dose antibiotics on dextran sodium sulfate (DSS) colitis. (**a**) Study design: 3-week weanling male C57BL/6 mice were give low-dose antibiotic treatment for 2 weeks, followed by a 1-week wash-out phase and then challenged with DSS. (**b**) Body weight variation relative to baseline of mice challenged with DSS. Data were pooled from 4 independent experiments. (n = 17–23/group). (**c**) Intestinal bleeding score changes of mice after DSS challenge. Data were pooled from 2 independent experiments (n = 12–13/group). (**d**) Histologic score of one representative experiment based on hemotoxylin and eoxin (HE) staining. Mice were sacrificed on day 9 (n = 5–6/group). (**e**) HE staining of distal colon harvested on day 9 of DSS treatment. Each panel is representative of tissue from at least 5 mice. C: control, P: penicillin, E: enrofloxacin, M: metronidazole. ^*^P < 0.05, ^**^P < 0.01, one-way ANOVA. Symbols represent mean ± SEM.

Early life after weaning is considered a critical window for both the development of gut microbiota and the immune system^15^. Here we used a mouse DSS-induced colitis model to examine whether perturbed microbiota may alter the susceptibility to IBD. Before DSS treatment, mice were exposed to low-dose penicillin, metronidazole or enrofloxacin respectively in drinking water for 2 weeks, followed by a 1-week wash-out phase (Fig. 1a). After DSS treatment, colitis symptoms occurred among all groups, but penicillin pre-treated mice exhibited a significantly smaller decline of bodyweight, which was most obvious on day 8 (Fig. 1b). The penicillin group also seemed to have a smallest intestinal bleeding score, though it was not statistically significant (Fig. 1c). Histological analysis further confirmed the disparity of tissue damage between groups. Crypt loss and leukocytes infiltration were common in all groups but mice in penicillin group seemed to preserve more crypts (Fig. 1d,e). Metronidazole pre-treated mice, however, displayed more severe submucosal swelling when compared with others. In all, low-dose penicillin exposure in weanling mice seemed to play a protective role against DSS colitis. Metronidazole and enrofloxacin, on the other hand, failed to yield the same effects.

### Low-dose penicillin treatment suppresses *Il17* expression and ileal Th17 differentiation

**Figure 2:**
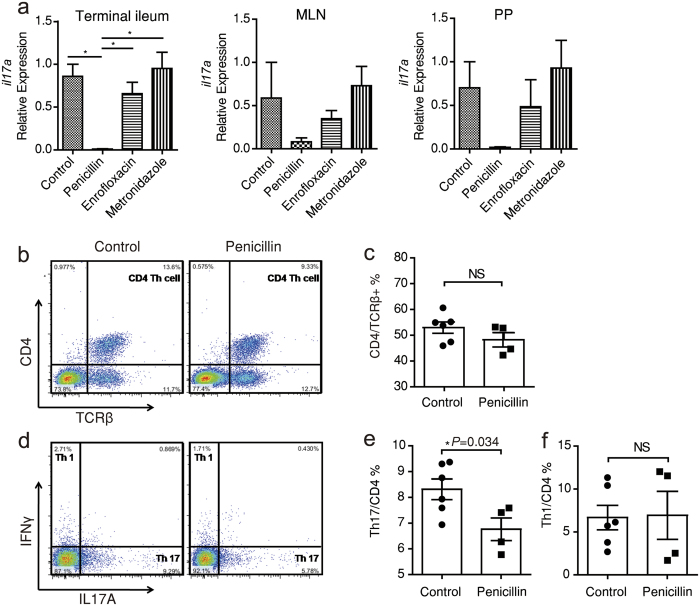
Low-dose penicillin's impact on *Il17* expression and Th17 cell differentiation. (**a**) Relative expression of *Il17a* in indicated tissues after 2-week treatment quantified by qPCR (n = 3/group). (**b**) TCRβ^+^CD4^+^ Th cell count in small intestine lamina propria (SI-LP) measured by flow cytometry. (**c**) Mean ± SEM indicating the proportion of CD4^+^ Th cells in TCRβ^+^ T cells from (**b**). (**d**) Intracellular staining of SI-LP mononuclear cells for IL-17A and IFNγ after stimulation by phorbol-12-myristate 13-acetate (PMA) and ionomycin. Plots were gated on CD4^+^ cells. (**e**,**f**) The proportion of Th1 and Th17 subsets from (**d**). Data of b-f are from mice sacrificed 1 week after treatment cessation. ^*^P < 0.05, one-way ANOVA (**a**), Student's *t*-test (**c**,**e** and **f**).

Help T (Th) cells play an important role in orchestrating inflammation in both chemically induced colitis and human IBD. The protective role of penicillin pre-treatment might be due to an immune fingerprint on T cell differentiation. So we next measured the gene expression of key cytokines of different Th cells in terminal ileum tissues, mesenteric lymph nodes (MLNs) and Peyer’s patches (PPs). Several cytokines were expressed differently under treatments, but penicillin decreased *Il17a* mRNA level in all three tissues consistently while metronidazole and enrofloxacin didn’t (Fig. 2a). *Il17f* expression in terminal ileum was also down-regulated by penicillin (Supplementary Fig. S1). IL-17 is a group of pro-inflammatory cytokines implicated in host defensive against infection. They are mainly produced by Th17 cells and these cells mostly differentiate in small-intestinal lamina propia (SI-LP)^16^,^17^. So we next examined if differentiation of SI-LP Th17 cells was interfered by penicillin. It turned out there were fewer Th17 cells residing in SI-LP after 2-week penicillin treatment (Supplementary Fig. S2) and the disparity still existed one week after penicillin cessation (Fig. 2d). As CD4+ Th cells and Th1 cells remained rather intact (Fig. 2b,c and f), the lack of Th17 cells is not due to an over-all suppression of T cells but a specific inhibition.

### Low-dose penicillin imposed transient and small changes to diversity and structure of fecal bacteria community

**Figure 3:**
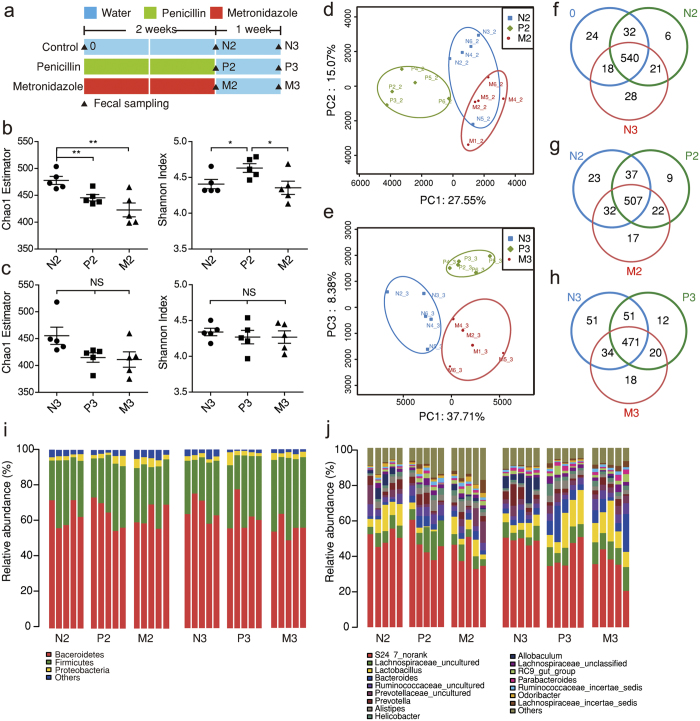
Transient and small alterations of gut microbial structure by low-dose penicillin. (**a**) Study design: Three-week weanling male C57BL/6 mice were treated with low-dose penicillin, metronidazole or regular water as control, respectively. Fecal samples were collected on baseline, week 2 and 3 (n = 5/group). (**b**,**c**) Chao1 richness estimator and Shannon diversity index of gut microbiota from samples collected on week 2 (**b**) and week 3 (**c**) after rarefying the sequencing depth. (**d**) Principle component analysis (PCA) plot of week-2 samples along principle component (PC) 1 and 2, which explained 27.55% and 15.07% of the total variance, respectively. (**e**) PCA plot of week-3 samples along PC1 and PC3, explaining 37.71% and 8.38% of the variance. (**f**–**h**) Venn diagrams showing shared OTUs among groups of different treatment and sample collecting time. (**i**,**j**) Microbial composition at phylum level (**i**) and gunus level (**j**). Data are expressed as mean ± SEM. ^*^P < 0.05, ^**^P < 0.01, ^NS^: not significant, one-way ANOVA.

In order to understand the gut microbial profile under low-dose antibiotic pressure, we applied 16S rRNA gene analysis of fecal bacteria from mice treated with regular water, low-dose penicillin or metronidazole, the latter two with disparate inflammation phenotypes and *Il17* expression. High-throughput sequencing produced 1842370 valid reads (with 48483 ± 14751 per sample) from 38 samples collected respectively at baseline, 2 weeks after antibiotic treatment and 1 week after treatment cessation (Fig. 3a). The reads were delineated into 688 operational taxonomy units (OTUs) at the similarity cut-off of 97%. The Good’s coverage index is 0.998 ± 0.0001, indicating an adequate sequencing depth, which is further proved by rarefaction analysis (Supplementary Fig. S3).

Estimators of alpha-diversity were calculated after rarefying the sequence depth to 19345 reads per sample, in order to avoid bias (Supplementary Fig. S3). According to Chao1 and Shannon index, both low-dose penicillin and metronidazole decreased bacterial richness in 2 weeks, while penicillin on its own increased bacterial diversity (Fig. 3b), which was similar to a previous study^12^. The impact of both antibiotics on alpha-diversity was transient, as the difference became insignificant one week later (Fig. 3c).

In order to preserve data from rare species, which may play important roles in gut homeostasis^18^, we used unrarefied sequences for other analysis in our study. Principle component analysis (PCA) revealed low-dose penicillin caused unique structural changes of fecal microbiota during and after treatment, as was shown by clustering of samples in plots (Fig. 3d,e). However, Venn diagrams showed the majority of OUTs were preserved and shared among groups under low-dose antibiotic treatment (Fig. 3f–h). The fecal microbiota were mainly composed of Bacteroidetes and Firmicutes in all groups, followed by far less abundant Proteobacteria, Candidate division TM7 and Actinobacteria (Fig. 3i). On genus level, low-dose treatment only caused small shifts of microbiota composition (Fig. 3j), compared with obvious dysbiosis induced by large-dose antibiotics in previous reports^8^,^9^.

### Key bacterial alterations under low-dose penicillin treatment

**Figure 4:**
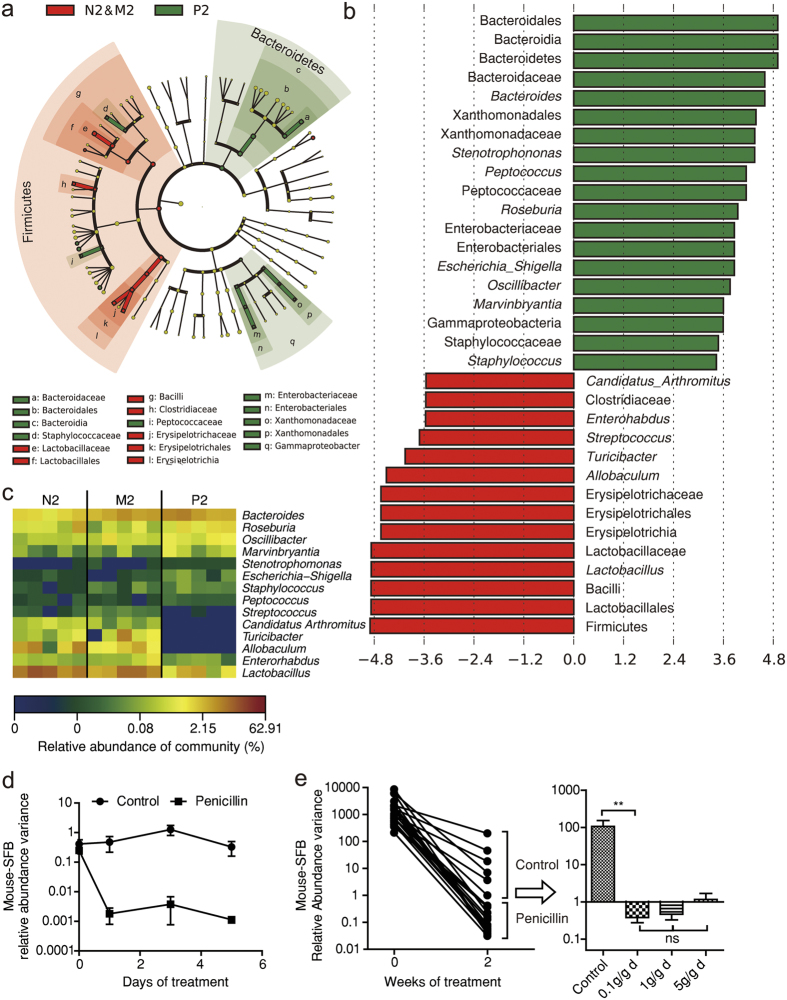
Key bacterial alterations under low-dose penicillin treatment. (**a**) Cladogram generated by LEfSe analysis showing enriched taxa in 2-week feces from control/metronidazol super-group (red) and penicillin group (green). (**b**) LDA scores of enriched taxa from a. (**c**) Heatmap showing relative abundance of key genera generated by LEfSe anaylsis. (**d**) Variance of segmented filamentous bacteria (SFB) relative abundance after penicillin treatment (1 μg/d · g bodyweight) quantified by qPCR. (**e**) SFB abundance changes under different levels of low-dose penicillin treatment. Data in d and e are expressed as relative fold difference to one of the samples and expressed as mean ± SEM. ^**^P < 0.01, ^NS^: not significant, one-way ANOVA.

In order to clarify which bacteria might be responsible for low-dose penicillin’s unique impact on intestinal inflammation, we conducted the LEfSe analysis for microbial biomarker discovery. By testing both the consistency and effect size of difference in bacteria abundance between groups, LEfSe suggests key bacteria at different taxonomy levels^19^. According to our experimental results, we combined control with metronidazole group into a super-group (each as a subgroup), as was opposed to penicillin group. According to the results, the key alteration caused by 2-week low-dose penicillin treatment was a decrease of *Enterohadus, Lactobacillus, streptococcus, Candidatus Arthromitus, Allobaculum*, and *Turicibacter*; and an increase in *Bacteroides, staphylococcus, Marvinbryantia, Roseburia, Peptococcus, Oscillibacter, Escherichia-Shigella* and *Stenotrophomonas*. At phylum level, low-dose penicillin treated mice featured a decrease of Firmicutes and an increase of Bacteroidetes and Gammaproteobacteria (Fig. 4a–c). One week after penicillin cessation, the disparity between two groups became less prominent, as was indicated by a smaller number of biomarkers at all levels. The key alterations in penicillin treated mice included a decrease in *Prevotella, Gemella, Ruminococcaceae, Candidatus Arthromitus, Allobaculum, Turicibacter* and *Erysipelotrichaceae*; and an increase in *Gordonibacter, Alistipes, Rikenella, Streptococcus* and *Marvinbryantia* (Supplementary Fig. S4).

Some of the key bacteria are potentially associated with IBD, such as *Prevotella*^20^,^21^,^22^, *Gemella*^20^ and *Erysipelotrichaceae*^23^. Among them, *Candidatus Arthromitus*, or segmented filamentous bacterium (SFB), has been known for its special immune-stimulating capacity for years^24^. Due to the limitation of culturing technique, we conducted PCR to investigate the bacteria’s distribution among mice. Most mice upon purchase were SFB-positive, while some were SFB-free. After one day’s treatment of penicillin, the load of SFB dropped dramatically (Fig. 4d). We also sought to define the minimum dose of penicillin for SFB eradication and found as little as 0.1 μg/d · g bodyweight of penicillin (equals to 0.6 mg/L penicillin in drinking water) had the same effect with higher dosages (Fig. 4e).

### Penicillin’s suppression of *Il17* expression and Th17 differentiation is SFB dependent

**Figure 5:**
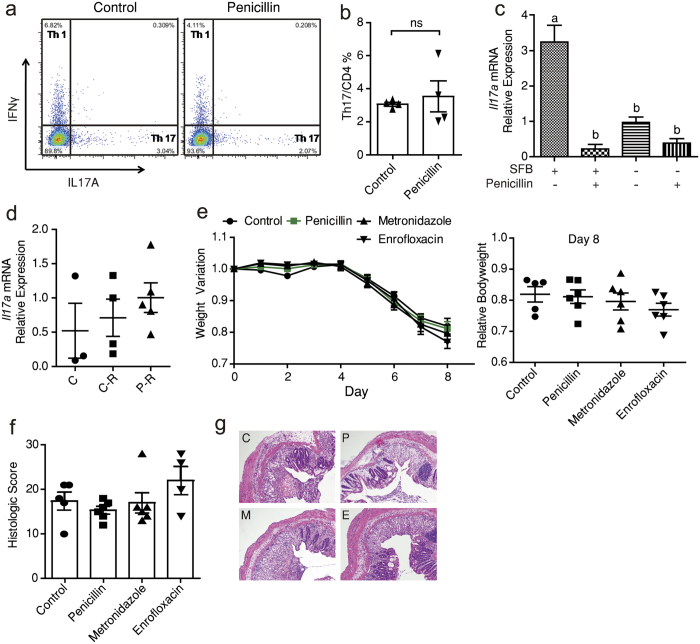
Penicillin's effects depend on the eradication of segmented filamentous bacteria (SFB). (**a**) 9-week-old adult mice were treated with low-dose penicillin or regular water as control for 2 weeks. Small intestine lamina propria (SI-LP) mononuclear cells were isolated and stimulated with phorbol-12-myristate 13-acetate (PMA) and ionomycin. Plots show IL-17A^+^ and IFNγ^+^ cells counted by flow cytometry and are gated on CD4^+^ cells. (**b**) Statistics of a. Data are expressed by mean ± SEM. ^ns^: not significant. (**c**) Three-week-old SFB-positive or SFB-negative weanling mice were treated with low-dose penicillin or regular water as control for 2 weeks (n = 5–6/group). Relative expression of *Il17a* in terminal ileum was measured by qPCR. Groups with different letters are significantly different at *P* < 0.05 tested by ANOVA. (**d**) Expression of *Il17a* in Peyer's patches of mice receiving 3-week gut microbiome manipulation described in methods (n = 3–5/group). Data are representative of 2 independent experiments. C: control mice without any interference, C-R: Recipient of control mice's microbiome, P-R: Recipients of penicillin-treated mice's microbiome. (**e**) Body weight variation of SFB-free mice challenged with dextran sodium sulfate (DSS) (n = 5–6/group). The study design was the same with Fig. 1a. (**f**) Histologic score of mice from e based on hemotoxylin and eoxin (HE) staining. Mice were sacrificed on day 8. (**g**) HE staining of the distal colon. Each panel is representative of tissue analyzed in (**f**). C: control, P: penicillin, E: enrofloxacin, M: metronidazole. ^ns^: not significant, one-way ANOVA (**c**–**f**), Student's *t*-test (**b**).

According to previous studies, adhesive mouse-specific SFB plays a crucial role in Th17 differentiation^16^,^17^,^25^,^26^. So we sought to clarify if eradication of SFB is responsible for penicillin’s effect. SFB’s colonization occurs at weaning, and declines in an age-dependent manner^27^ (see also Supplementary Fig. S4). At week 9, all mice became free of SFB^27^. We then treated 9-week-old mice with low-dose penicillin, and found no difference in SI-LP Th17 count between penicillin and control groups (Fig. 5a,b). In another group of SFB-free weanling mice, penicillin caused a much less and insignificant drop of *Il17a* expression (Fig. 5c). To sum up, the decrease of Th17 only occurred in SFB-positive mice. Moreover, ileal Serum amyloid A (SAA), which is responsible for SFB’s immune-stimulating capacity^28^,^29^, was also suppressed by penicillin (Supplementary Fig. S5).

However, there is still a possibility that penicillin’s effect partly relies on the enriched taxa after treatment, apart from the suppressed ones. To test this, we transplanted bacteria from caecal content of antibiotic treated mice, to 3-week SFB-positive weanling mice through gavage. The recipient mice failed to show a trend of *Il17a* suppression (Fig. 5d). This further strengthened the idea that it was not the enriched genera, but the decreased genera, especially SFB, which were responsible for the penicillin-induced IL-17 inhibition. This is in consistency with a recent theory^10^.

We also induced DSS colitis in a group of SFB-free mice. This time, the weight variance and histology showed similar degrees of inflammation among different groups (Fig. 5e–g).

## Discussion

In the present study, we examined the impact of low-dose antibiotic pre-treatment on intestinal inflammation with a mouse model. Among the tested antibiotics, penicillin yielded an unexpected protective effect against DSS colitis. A 2-week penicillin treatment led to a prominent drop of *Il17a* gene expression in intestinal immune tissues and a decrease of Th17 cells in SI-LP. We also found the effect of low-dose penicillin was associated with the eradication of commensal bacteria SFB, which is highly sensitive to this antibiotic.

Penicillin was the first antibiotic isolated and used against bacterial infection. The bacteriocidal effect depends on the inhibition of DD-transpeptidases by β-lactam rings, which causes a disbalance between building and breaking down of bacterial cell walls. Penicillin and other β-lactams are among the top antibiotics consumed worldwide^30^,^31^,^32^. A recent meta-analysis pooling risk-factor studies of IBD in western countries concluded that penicillin is the only antibiotic that wasn’t associated with IBD onset, while fluoroquinolones and metronidazole were most strongly related with IBD^3^. In our study, a 2-week pre-treatment of enrofloxacin or metronidazole failed to aggravate later DSS colitis, while penicillin turned out to be protective. This further indicates the complexity of the association between antibiotics and IBD pathogenesis. Some basic science researches have revealed penicillin’s extra-anti-infective effects on hosts through the modulation of gut microbiota. Zhang *et al*. reported that penicillin therapy could inhibit neutrophil aging and further alleviate inflammation-related tissue damage of sickle cell disease or endotoxin-induced septic shock^33^. Cox *et al*. first discovered that low-dose penicillin yielded a lasting growth promotion effect in mice when given in early life^12^. Our results enhance the knowledge of low-dose penicillin, showing only a 2-week short-period intervention could leave animals in a less inflammation-favoring state.

Gut microbiota have long been recognized as key factors in IBD pathogenesis. Many IBD-associating genes and pathways have roles in microbial defense and intestinal immune homeostasis^34^. A number of environmental risk factors of IBD can also be triggers of gut microbiota alterations^35^. There are several specific microorganisms considered as possible causative agents of the disease, such as *Mycobacterium avium* subspecies paratuberculosis (MAP), and *Escherichia coli*^36^. With the help of low antibiotic dose, which didn’t cause severe dysbiosis and suppressed only a few genera, we were able to define a group of key bacteria and highlight the importance of SFB. SFB, or *Candidatus Arthromitus* as the genus name, is a commensal bacterium mainly inhabits the small intestine, forming attachment sites on epithelia cells^37^. SFB genome sequencing revealed a lack of ability of biosynthesis, indicating that its life style is highly dependent on host cells^38^. SFB had been unculturable, until recently, a team developed an SFB-host cell co-culturing system^26^. Previous studies with electron microscopes found prominent morphology changes of SFB within 3–5 hours after the hosts were given a large dose of penicillin, inferring its sensitivity to penicillin^39^. We think the high demand for cell wall construction for numerous intracellular septa during a rapid life cycle is the reason why SFB could be eradicated with such a low dose of penicillin in our experiments^26^. Low-dose metronidazole, while decreasing SFB load, was not able to achieve a complete wipe-out. This makes penicillin a specific and ideal tool for SFB manipulation.

The importance of SFB lies on its unique ability to elicit physiological inflammation. When adhering to epithelial cells, SFB is able to promote Th17 differentiation, IgA secretion and antimicrobial peptide production^26^. Though commonly detected in various young animals as a commensal^27^, SFB is reported to exacerbate autoimmune encephalitis and arthritis in mouse model^40^,^41^. In the case of intestinal inflammation, SFB could induce colitis in SCID mice transferred with CD4^+^CD45RB^high^ T cells, when co-colonizing with a cocktail of other bacteria^42^. SFB was also found highly coated with IgA, which turned out as a marker of colitogenic microbes^23^. Moreover, though not usually found in grown-up animals or adult human, there are case reports providing evidence that SFB could be detected at certain inflammatory sites both in UC and CD patients^43^,^44^. Our finding reinforces the notion that SFB, though not able to cause intestinal inflammation on its own, could be part of the pathogenesis^42^.

SFB’s pro-inflammatory potential was mainly attributed to its ability to inducing IL-17-producing cells, including Th17. Similar to a previous report^12^, we found down-regulation of Th17 cells in SI-LP by low-dose penicillin. Th17 cells are an important part of the immune system. Researchers have discovered an enrichment of balancing selection in genes related to IL-17 production^45^. This implies a need for balance between microbial defense and inflammation during evolution^45^. Under normal condition, Th17 cell can promote neutrophil function and antibacterial peptide production, but they may also trigger autoimmune diseases including IBD, rheumatoid arthritis, psoriasis and multiple sclerosis^46^. Several genes in IL-17/IL-23 pathway have been observed as IBD risk loci, including IL-23R, IL-22, RORC, IL-21, Stat3 and AHR^34^,^47^. SFB is recently reported to induce Th17 differentiation through activation of an IL-23R/IL-22 circuit^28^. Thus, SFB and IL-17/Th17 are closely associated with IBD pathogenesis. However, whether the alleviation of DSS colitis was caused by Th17 inhibition is still to be elucidated. IL-17, though usually viewed as a pro-inflammatory cytokine, plays a rather complicated role in human IBD as well as experimental colitis. While the dual blockade of IL-17A and IL-17F attenuated intestinal inflammation^48^. Th17-deficient mice presented even more severe DSS colitis than wild type ones^49^. While antibodies targeting IL-17A proved ineffective in treating CD patients^50^, some small-molecule inhibitors have shown efficacy in clinical trials^51^,^52^. Overall, there is a complex and delicate balance between the protective and pathogenic roles of IL-17 and Th17 cells. Targeting IL-17 during a more acute phase of intestinal inflammation, or even as a prophylactic therapy ahead of disease flare, could be a more rational approach^53^. Here in the present study, why did the inhibition of IL-17 yield anti-inflammatory results? We put forward several explanations. First of all, penicillin achieved a dual suppression of *Il17a* and *Il17f* expression, the latter as a more pro-inflammatory cytokine. Secondly, penicillin-treated mice were not deprived of all Th17 cells, which may preserve more protective capacity in compare with the case of IL-17 knock-out mice. Thirdly, the pre-treatment had put mice in a low-Th17 state before DSS colitis, mimicking a prophylactic therapy. DSS colitis is featured with massive neutrophil infiltration in intestinal tissue. Th17 cells and neutrophils can reciprocally recruit each other in inflamed sites, forming a vicious cycle^54^. When Th17 cell differentiation is inhibited by penicillin, the vicious cycle can be less strong. Overall, we suggest that the down regulation of IL-17 and Th17 could be responsible for penicillin’s protective effects through eradication of SFB.

There are several limitations in our research. We failed to conduct further experiments with other bacteria that also had biological importance according to LEfSe analysis, especially Turicibacter and Allobaculum, of which behavior was highly similar to SFB under penicillin pressure. Secondly, we used conventional mice to achieve a more real-life results with a more diverse microbiome, yet at the possible risk of inter-study variations. Further researches with germ-free or gnotobiotic mice together with new culturing methods for key bacteria are needed for better control of the composition of gut microbiota. Thirdly, we applied DSS colitis model in the study, while chemically induced inflammation cannot fully recapitulate human IBD. However, there is another report in which the authors directly examined the colitogenic effect of SFB-containing bacterial cocktails with a T-cell transferred colitis model^42^. Interestingly, they found that SFB together with a cocktail of bacteria were effective to trigger intestinal inflammation, which is very supportive of our conclusion. Genetically modified mice should be used in future to elucidate the compound effects of SFB and IBD risk genes especially in IL-17/IL-23 pathway.

In conclusion, through a highly specific manipulation of sensitive bacteria, a short-term treatment of low-dose penicillin has shown its potential to interfere with development of intestinal immune system in early life. It may partly answer the epidemiologic question why different antibiotic exposure imposed distinct risk on IBD onset. Our study may further indicate the possibility of achieving a favorable immune state among a certain group of patients with IBD, or other autoimmune diseases, by fine-tuning the gut microbiota.

## Methods

### Animals and antibiotic treatment

Three-week-old C57BL/6 mice were purchased from SLAC laboratory (Shanghai, China) and maintained under conventional barrier conditions with a 12-hour light/dark cycle in Shanghai Jiao-Tong University, School of agriculture and biology, or the experimental animal centre of Tongji University. All cages and water were autoclaved prior to use, with bedding and chow irradiated. Low dose antibiotics were supplied in drinking water *ad libitum* at the concentration of 6.67 mg/L, which equals to 1 μg/g bodyweight according to previous reports^12^,^55^. Antibiotic intervention began on the next day of animal receipt and lasted for 2 weeks. In order to maintain the concentration of antibiotics against degradation, penicillin and metronidazole were renewed every day, with enrofloxacin every three days^56^,^57^,^58^. All animal experimentations were approved by the scientific research ethical committee of Renji Hospital affiliated to Shanghai Jiao-Tong University. Procedures involving animals and their care were conducted following NIH guide for the care and use of laboratory animals (eighth edition).

### Induction of colitis and determination of clinical scores

Acute colitis was induced with 2.5% (w/v) DSS (molecular weight 36000–50000, MP Biomedicals, US) dissolved in drinking water for 7 days. As described in a protocol, newly-made DSS solution was given on day 0, day 2 and day 4, followed by regular water on day 7 until termination of the experiment on day 8–10^59^. DSS solution was always given 1 week after antibiotic cessation, in order to exclude the direct effect of antibiotic residue in animals. Body weight was determined every day, as well as stool consistency and rectal bleeding state. Occult blood was assessed with a urine-fecal occult blood test kit. An established scoring system for intestinal bleeding was used as an index of disease activity^59^.

### Hemotoxylin and eoxin (HE) staining and histologic analysis

Following euthanasia on day 9, 0.5 cm of distal colons were dissected, washed in saline and fixed in 4% paraformalin for 24 h at 4 °C. The tissues were then embedded in paraffin and sectioned to 4 μm thickness. HE staining was performed by an autostaining machine following standard protocols, while images acquired by an Olympus BX43 microscope. Based on a previously described scoring system, each tissue was assigned with four scores for epithelial damage, inflammatory infiltration of mucosa, submucosa and muscularis/serosa. The scores were multiplied with an index according to the extent of tissue damage^60^.

### Microbial community analysis by 16S rRNA gene sequencing using Illumina technology

Mouse feces were collected into autoclaved eppendorf tubes and stored at −80 °C after snap frozen in liquid nitrogen. Fecal genomic DNA was extracted with OMGA-soil DNA kit following the manufacturer’s instruction. Hypervariant region V3-V4 of bacterial 16S rRNA gene was amplified with the primer 338F (5′-barcode-ACTCCTACGGGAGGCAGCA-3′) and 806R (5′-ACTCCTACGGGAGGCAGCA-3′) by PCR. PCR reactions were carried out in 20 μL mixtures containing 10 ng of template DNA, 0.8 μL of each primers (5 μM), 0.4 μL of FastPfu polymerase (TransGen, Beijing), 2 μL of 2.5 mM dNTPs, and 4 μL of 5* FastPfu buffer. Reaction condition was 3 mins at 95 °C, and then 27 cycles of 30 s at 95 °C, 30 s at 55 °C and 45 s at 72 °C, and a final extension phase of 10 mins at 72 °C. Products were purified with AxyPrep DNA Gel Extraction Kit (Axygen Bioscience, CA, USA) and quantified with Quantifluor-ST (Promega, US). Purified amplicons were sequenced on an Illumina MiSeq platform at Majorbio Bio-pharm Technology Co. Ltd according to the standard protocols.

### Processing of sequencing data

Raw fastq files were quality-filtered using QIIME^61^ (version 1.17). Reads which could not be assembled were rejected. Operational taxonomic units (OTUs) were clustered by UPARSE^62^(version 7.1 http://drive5.com/uparse/) with 97% similarity and chimeric sequences were identified and removed using UCHIME. The taxonomy of each sequence was analyzed by RDP Classifier against Silva (SSU115) 16S rRNA database with 70% confidence threshold^63^,^64^. Rarefaction analysis was performed by Mothur and alpha-diversity indexes were compared using rarefied data. Principle component analysis (PCA) plot and Venn diagram were implemented by R programming language. Significant changes in relative abundance of microbial taxa were detected by linear descriminant analysis effect size (LEfSe) method^19^.

### Segmented Filamentous Bacteria (SFB) quantification by real-time PCR

Fecal genomic DNA was extracted by Tiangen Fecal Genomic DNA Extraction Kit under the manufacturer’s instruction. Quantitative PCR was carried out by Applied Biosystems 7500 with SYBR Premix Ex Taq quantitative PCR Kit using the universal bacteria primers (5′-ACTCCTACGGGAGGCAGCAGT-3′ and 5′-ATTACCGCGGCTGCTGCG-3′)^17^, mouse-specific SFB primers (5′-TGAGCGGAGATATATGGAGC-3′ and 5′-CATGCAACTATATAGCTATATGCGG-3′) and rat-specific SFB primers (5′-TGAAGCGGAGGTAGATGGA-3′and 5′-GCAACTATATAGCTGTATGCGG-3′)^29^. PCR reactions were performed as indicated by Takara protocol. The results were expressed as fold changes of relative abundance variation of SFB. Each mouse was tested for SFB upon arrival.

### Tissue RNA extraction and quantification of gene expression with real-time PCR

Tissues were stored in RNAlater (Qiagen) at −20 °C upon harvesting. Total RNA was isolated using TRIzol (Invitrogen, CA, USA) according to the manufacturer's instruction. Quantification real-time PCR was performed with SYBR Premix Ex Taq quantitative PCR Kit in a StepOnePlus apparatus. Results were analyzed with ΔΔCT method and normalized to the gene *Gapdh*. Primers used in the experiment are included in Supplementary Table S1.

### Small intestinal lamina propria lymphocyte isolation and flow cytometry

Mouse ilea were collected at sacrifice. Peyer's patches were carefully excised. Ilea were then cut longitudinally and washed with saline to fully remove intestinal content. Before further processing, tissues were stored at 4 °C in complete 1640 containing 5% fetal bovine serum (FBS; Gemini bio, US), 200 U/ml penicillin, 200 μg/ml streptomycin, 10 μg/ml amphotericin B and 100 μg/ml gentamycin. Mucus and epithelium were removed by two 20 mins washes at 37 °C in calcium-free PBS containing 5 mM dithiothreitol (DTT), 5 mM EDTA, 5% FBS, 200 U/ml penicillin, 200 μg/ml streptomycin, 10 μg/ml amphotericin B and 100 μg/ml gentamycin. The remaining tissues were excised into fine pieces and washed in PBS, and then digested in complete 1640 containing 1% collagenase (Worthington Biochemical, US), 1% DNase (Sangon, Shanghai, China) and 2% FBS, at 37 °C and 150 rpm, for 20 mins. After a 7 s vortex for adequate isolation, cells were eluted by passing through a 70-micron strainer, washed with PBS and resuspended in 40% Percoll (GE Healthcare, US) in complete 1640. Cell suspension were laid onto 80% Percoll and centrifuged at 750 g for 15 mins at 4 °C without braking. Lamina propria mononuclear cells (LPMCs) were collected at the interface and used for further experiment. For flow cytometry analysis, LPMCs were stimulated with 50 ng/ml phorbol-12-myristate 13-acetate (PMA; Sigma, US) and 750 ng/ml ionomycin (Sigma) for 4 hrs at 37 °C in the presence of monensin (BD biosciences, US). Following stimulation, cells were permeablized by Perm/Wash buffer set (BD biosciences) under the manufacturer's instruction and stained with anti-CD4-FITC (BD biosciences), anti-TCRβ-PE (BD biosciences), anti-IL-17A-PE (BD biosciences) and anti-IFNγ-APC (ebioscience). Cells were acquired on a Fortessa flow cytometry and analyzed with Flowjo 7.6.1 software.

### Manipulation of gut microbiome by gavage

Cecal content from donor mice were taken after sacrifice, suspended in 10 volumes (w/v) 20% glycerol/PBS solution and stored at −80 °C. Upon use, frozen suspension was thawed and centrifuged at 4 °C for 8 mins (8000 g). The pellet was resuspended with the same volume of PBS. 100 μL of the suspension was given to each recipient by oral gavage using 22 ga * 25 mm plastic feeding tubes. Gavage was performed every week for 3 weeks, in order to maintain a stable microbial composition. No adverse effect was observed during experiments.

### Statistical analysis

Data are presented as mean ± SEM. Shapiro-wilk test and Levene's test were used for determination of normality and homogeneity of variance, respectively. According to distribution of data, comparisons between different groups were carried out with one-tailed unpaired *t*-test (between 2 groups), one-way ANOVA corrected for multiple comparison with SNK tests (among more than 2 groups), or Wilcoxon rank sum test (for data unsuitable for *t*-test or ANOVA). Differences were considered statistically significant at *P* < 0.05. Analysis was performed with SPSS 19 software.

## Additional Information

**How to cite this article:** Jin, S. *et al*. Low-dose penicillin exposure in early life decreases Th17 and the susceptibility to DSS colitis in mice through gut microbiota modification. *Sci. Rep.* **7**, 43662; doi: 10.1038/srep43662 (2017).

**Publisher's note:** Springer Nature remains neutral with regard to jurisdictional claims in published maps and institutional affiliations.

## Supplementary Material

Supplementary Material
