# Supplementary material for: Low-dose penicillin exposure in early life decreases Th17 and the susceptibility to DSS colitis in mice through gut microbiota modification
Source: Sci Rep. 2017 Mar 8;7:43662. doi: 10.1038/srep43662 (PMC5341569; doi:10.1038/srep43662)
Supplement: Supplementary Material [file srep43662-s1.pdf]

Supplementary Material

Low-dose penicillin exposure in early life decreases Th17 and the susceptibility to DSS colitis in mice through gut microbiota modification

Shuang Jin, Di Zhao, Chenwen Cai, Dongjuan Song, Jun Shen, Antao Xu, Yuqi Qiao, Zhihua Ran* and Qing Zheng*

Division of Gastroenterology and Hepatology; Key Laboratory of Gastroenterology and Hepatology, Ministry of Health; Renji Hospital, School of Medicine, Shanghai Jiao-Tong University; Shanghai Institute of Digestive Disease; Shanghai Inflammatory Bowel Disease Research Center; 160 Pujian Road, Shanghai 200127, China

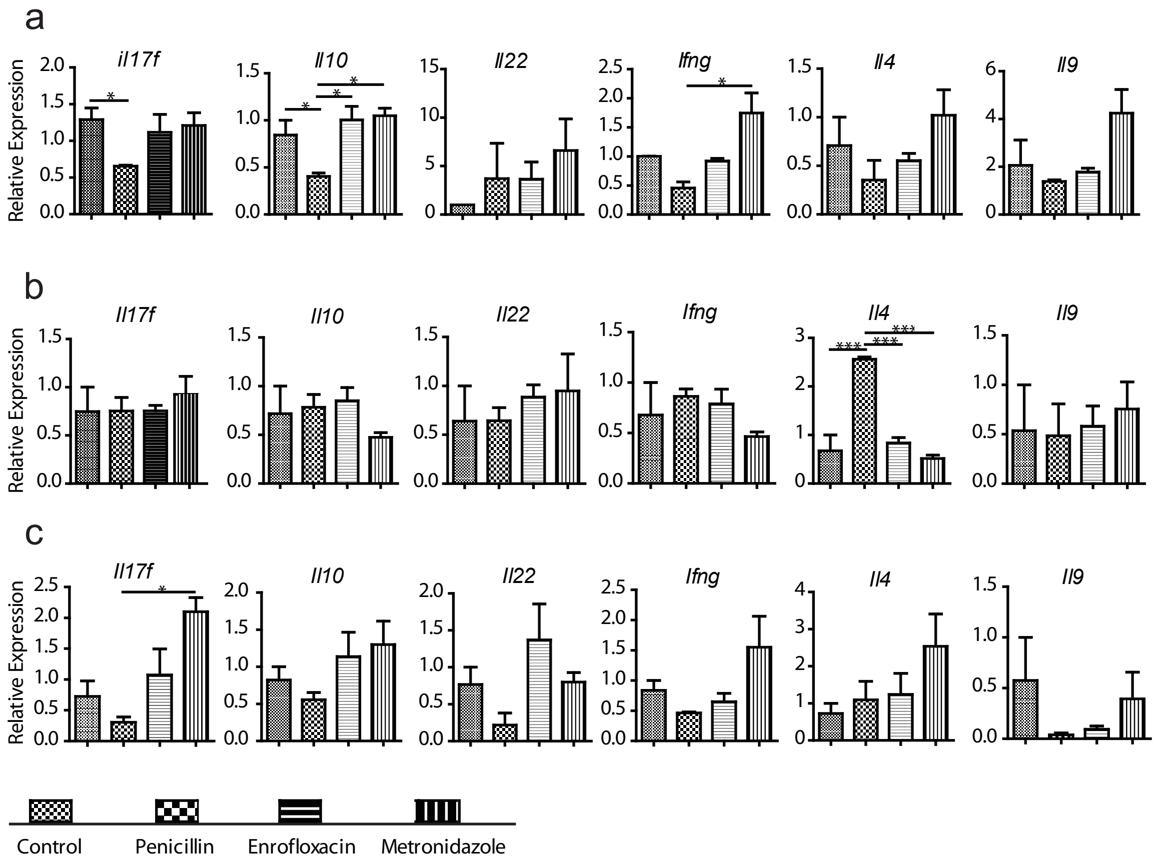

Supplementary figure S1: Low-dose antibiotics' impact on cytokine expression quantified by qPCR (n=3/group). Relative expression of indicated cytokine genes after 2-week treatment was measured in terminal ileum tissue (a), mesenteric lymph nodes (b) and Peyer's patches (c). Data are presented as mean ± SEM. *P<0.05, ** P<0.01, ***P<0.001, one-way ANOVA.

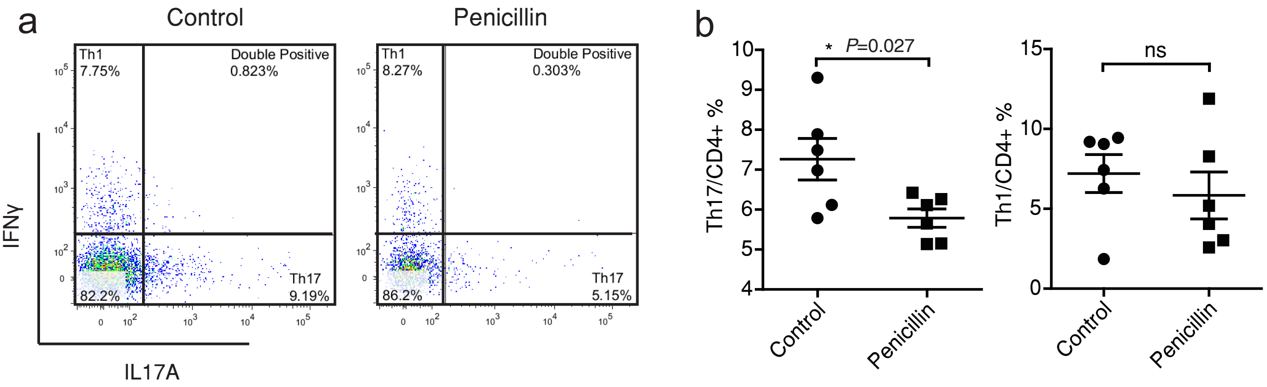

Supplementary figure S2: Two weeks' treatment of low-dose penicillin suppressed Th17 differentiation in SI-LP. (a) Intracellular staining of Small intestinal lamina propria (SI-LP) mononuclear cells for IL-17A and IFNγ after stimulation by PMA and ionomycin. Plots were gated on CD4+ cells. (b) Statistics of a. *:$P < 0.05$, Student's *t*-test.

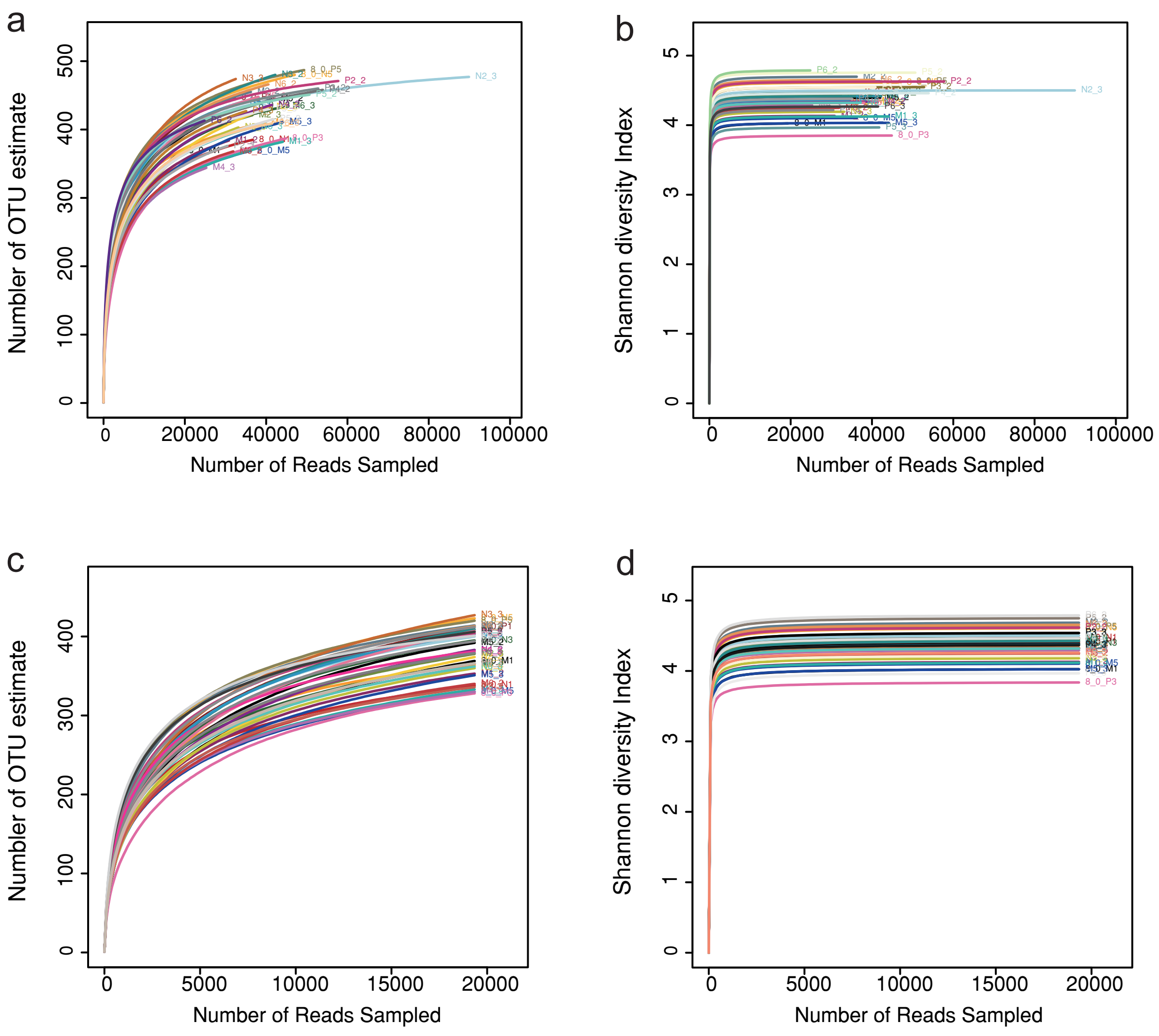

Supplementary figure S3: Rarefaction of samples. Rarefaction curves (a, c) and Shannon-Wiener curves (b, d) before rarefying of data (a, b) and after rarefying the sequence depth to 19345 reads per sample (c, d).

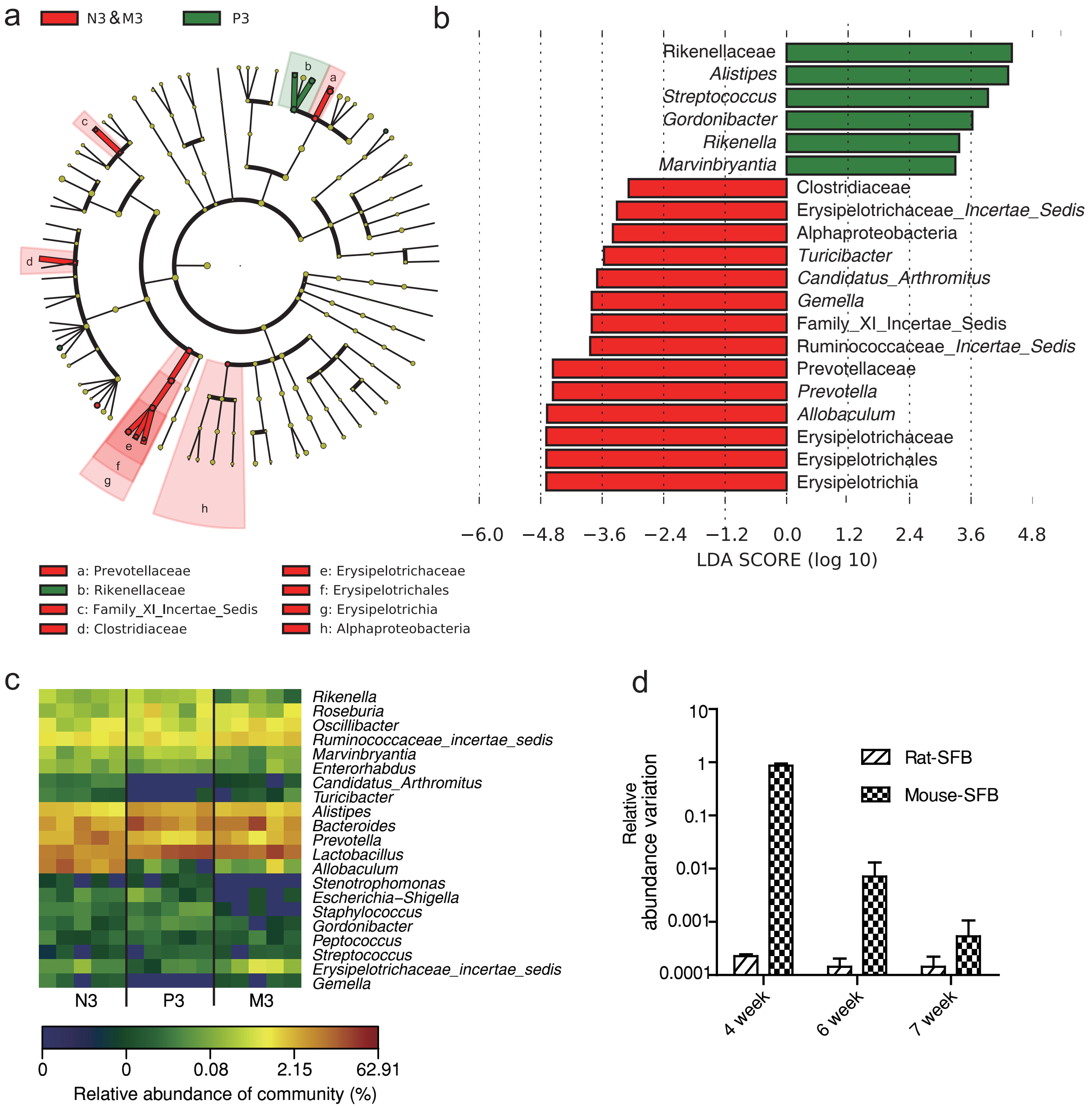

Supplementary figure S4: Key bacterial alterations after antibiotic cessation. (a) Cladogram generated by LEfSe analysis showing enriched taxa in control/metronidazol super-group (red) and penicillin group (green) 3-week feces. (b) LDA scores of enriched taxa from a. (c) Heatmap showing relative abundance of key genera generated by LEfSe anaylsis. (d) Rat-SFB and Mouse-SFB relative abundance quantified by qPCR in indicated age. Data in d are expressed as relative fold difference to one of the samples.

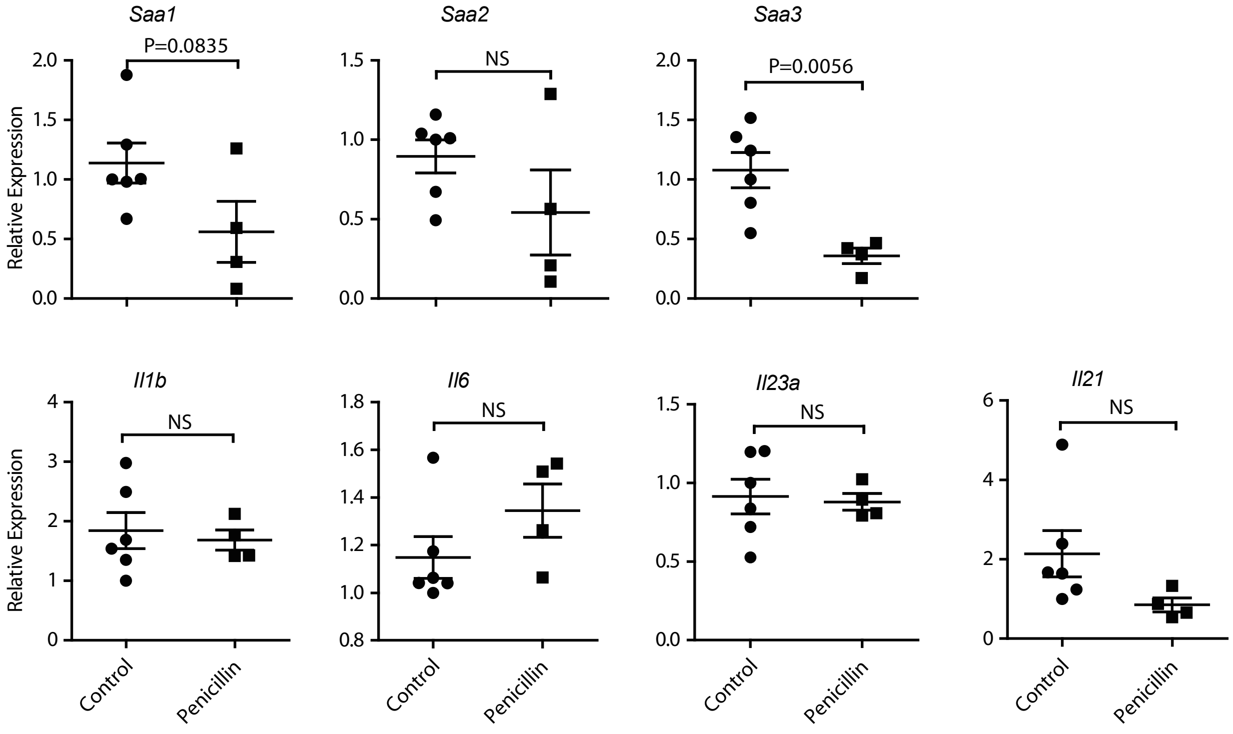

Supplementary figure S5: Relative expression of Th17-differentiation-related proteins in terminal ileum tissue. Data are represented as mean ± SEM. NS: not significant, Student's ***t***-test.

Primers used in the experiment:

Gene	Forward Primer	Reverse Primer
Gapdh	AGGTCGGTGTGAACGGATTTG	TGTAGACCATGTAGTTGAGGTCA
Saa3	TGCCATCATTCTTTGCATCTTGA	CCGTGAACTTCTGAACAGCCT
Saa2	TGGCTGGAAAGATGGAGACAA	AAAGCTCTCTCTTGCATCACTG
Saa1	GACAGAGGATGAAGCTACTCACC	CCTTGGAAAGCCTCGTGAAC
Il1b	GCAACTGTTCCTGAACTCAACT	ATCTTTTGGGGTCCGTCAACT
Il6	CCTACCCCAATTTCCAATGCTCTCC	GCATAACGCACTAGGTTTGCCGA
Il23a	CAGCAGCTCTCTCGGAATCTC	TGGATACGGGGCACATTATTTTT
Il21	GGACCCTTGTCTGTCTGGTAG	TGTGGAGCTGATAGAAGTTCAGG
Il9	TTGTGTCTCTCCGTCCCAAC	TGGTCAGCTGTAACAGTCCC
Il22	CAGCTCAGCTCCTGTCACATC	AGTTCCCCAATCGCCTTGAT
Il4	TCGGCATTTTGAACGAGGTC	TGGTGTTCTTCGTTGCTGTGA
Ifng	AGACAATCAGGCCATCAGCAA	TGTGGGTTGTTGACCTCAAACT
Il17a	ACCTCACACGAGGCACAAGT	GCAACAGCATCAGAGACACAGA
Il17f	AATCCTGGTCCTTCGGAGGG	GCCAACTTTTAGGAGCATCTTCT
Il10	CAGGACTTTAAGGGTTACTTGGGT	GCCTGGGGCATCACTTCTAC

Supplementary Table S1: Primers used in qPCR, related to Fig. 2, Supplementary Fig. S1 and Supplementary Fig. S5.
